# A transition from enemies to allies: how viruses improve drought resilience in plants

**DOI:** 10.1007/s44154-024-00172-y

**Published:** 2024-07-10

**Authors:** Ved Prakash, Veerendra Sharma, Ragunathan Devendran, Ramgopal Prajapati, Bilal Ahmad, Ritesh Kumar

**Affiliations:** 1 Department of Plant Pathology, Kansas State University, Manhattan, KS USA; 2 Division of Biology, Kansas State University, Manhattan, KS USA; 3 Martin-Luther University, Halle, Germany; 4 Boyce Thompson Institute, Ithaca, NY USA

**Keywords:** Drought, Virus, Resistance, Abiotic stress, Global warming, Crop, Productivity

## Abstract

Global crop production is severely affected by environmental factors such as drought, salinity, cold, flood etc. Among these stresses, drought is one of the major abiotic stresses reducing crop productivity. It is expected that drought conditions will further increase because of the increasing global temperature. In general, viruses are seen as a pathogen affecting the crop productivity. However, several researches are showing that viruses can induce drought tolerance in plants. This review explores the mechanisms underlying the interplay between viral infections and the drought response mechanisms in plants. We tried to address the molecular pathways and physiological changes induced by viruses that confer drought tolerance, including alterations in hormone signaling, antioxidant defenses, scavenging the reactive oxygen species, role of RNA silencing and miRNA pathway, change in the expression of several genes including heat shock proteins, cellulose synthase etc. Furthermore, we discuss various viruses implicated in providing drought tolerance and examine the range of plant species exhibiting this phenomenon. By applying current knowledge and identifying gaps in understanding, this review aims to provide valuable insights into the complex dynamics of virus-induced drought tolerance in plants, paving the way for future research directions and practical applications in sustainable agriculture.

## Introduction

Global warming has been identified as a significant factor contributing to the increase in drought occurrences, which in turn has a substantial impact on food productivity (IPCC 2021; Kumar et al. 2022; Janni et al. 2024). In the last 40 years, the percentage of drought affected land of our planet has doubled (Agency 2017). From 1998 to 2017, drought caused an enormous economic loss of $124 billion (Daniel et al. 2022). In the developing nations, drought caused $29 billion loss in agriculture from 2005–2015 (FAO 2018). Studies have shown that droughts have led to a reduction in global crop production, with historical records indicating a 10% decrease from 1964 to 2007 (Kim et al. 2019). Furthermore, it is predicted that under the global warming scenario, drought will continue to impact crop production globally, exacerbating food shortages (Zhao and Wang 2021). The potential impacts of drought on agricultural production are critical for ensuring global food security (Leng and Hall 2019). The implications of these findings are far-reaching, as global warming is not only projected to affect crop productivity but also crop quality, leading to economic losses (Masutomi et al. 2019; Chhaya et al. 2021). Additionally, the increasing occurrence of severe droughts is expected to double the rate of drought-induced yield losses in the largest warming scenario, further exacerbating the challenges faced by food production systems (Yu et al. 2018).

Plant viruses are obligate parasites and cause enormous crop losses to the growers posing significant challenges to global agriculture. (Hanssen et al. 2010; Gnanasekaran and Chakraborty 2018; Gnanasekaran et al. 2019a) There are several examples of devastating viruses affecting the yield and quality of fruits, vegetables and grains etc. (Gnanasekaran et al. 2019b, 2021; Moriones and Verdin 2020). Though, viruses are considered as devastating pathogens causing significant crop loss globally, however, research shows that viruses could also be beneficial under certain circumstances. Several studies have demonstrated that virus infections can trigger defense responses in plants, leading to increased tolerance to various abiotic stresses such as drought, salt, freezing, and heat stress (Kasuga et al. 1999; Koo et al. 2020; Augustine et al. 2022). For instance, it has been shown that virus infections can induce transgenerational tolerance to salt and osmotic stresses in plants (Hernández-Walias et al. 2022). Additionally, heat-killed *Tobamovirus* (RNA virus) *Tobacco mosaic virus* (TMV) has been found to increase abiotic stress tolerance in plants, suggesting a potential application in challenging growth environments (Augustine et al. 2022). Furthermore, the role of plant hormones, such as salicylic acid (SA) and jasmonic acid (JA), in mediating resistance to biotic stresses and tolerance to different abiotic stresses has been highlighted (Aguilar et al. 2017; Koo et al. 2020).

Viruses have been found to play a role in inducing drought tolerance in plants, offering potential solutions to mitigate the impact of drought on crop productivity. For instance, the first descriptions of a virus-dependent increase in plant tolerance to drought have been associated with RNA viruses such as *Bromovirus Brome mosaic virus* (BMV)*, Cucumovirus Cucumber mosaic virus* (CMV)*, TMV,* and *Tobravirus Tobacco rattle virus* (TRV) (Xu et al. 2008). Studies have shown that the interaction between *Potexvirus* (RNA virus) *Potato virus X* (PVX) and *Potyvirus* (RNA virus) *Plum pox virus* (PPV) can lead to improved tolerance to drought in *Nicotiana benthamiana* and *Arabidopsis thaliana*, suggesting that increased virulence exhibited by the synergistic interaction of these viruses may confer benefits in response to drought conditions (Aguilar et al. 2017). Wilting in *Begomovirus* (DNA virus) *Tomato yellow leaf curl Sardinia virus* (TYLCSV) infected tomato was slower and post-dehydration recovery was faster compared to healthy plants (Sacco Botto et al. 2023). In another study, C4 protein of TYLCSV has been found to prime drought tolerance in tomato through morphological adjustments, indicating the potential of viral proteins in enhancing plant resilience to drought stress (Pagliarani et al. 2022). Moreover, C4 protein of Begomovirus *Tomato yellow leaf curl virus* (TYLCV) also has been shown to provide tolerance to drought in tomato and *N. benthamiana* (Corrales-Gutierrez et al. 2020). These findings underscore the potential of viruses in conferring drought tolerance in plants. In addition, the role of RNA viruses such as CMV in inducing drought tolerance has been highlighted, with the CMV-encoded 2b protein interfering with abscisic acid (ABA) mediated signaling and inducing drought tolerance in *A. thaliana* (Westwood et al. 2013).

There are several mechanisms through which viruses can help their hosts mitigate drought stress, and these mechanisms vary depending on a specific virus-host system. A particular plant species can respond to drought stress differently when infected with different viruses, and a specific virus can cause different responses in different plant species (Ramegowda and Senthil-Kumar 2015). The mechanism of virus-induced drought tolerance depends on the virus strain and the host genotype. In this review, we discussed various mechanisms utilized by viruses to induce drought resilience in plants which could be helpful in developing/engineering crops with enhanced drought tolerance.

## Plant hormone mediated: Role of salicylic acid (SA) and abscisic acid (ABA)

Phytohormones plays a crucial role in plant responses to various stresses, including biotic and abiotic stresses. Research show importance of hormonal crosstalk during plant growth, development and stress responses (Yang et al. 2019; Mishra and Sarkar 2023). For example, during stress conditions or various developmental processes, crosstalk between JA, gibberellic acid (GA), cytokinin and auxin occurs (Liu and Timko 2021). Additionally, during several abiotic and biotic stresses, the crosstalk between ABA and SA has been observed which determines the outcome of plant-pathogen interactions (Cao et al. 2011). Moreover, it is the inter-hormonal crosstalk which has been demonstrated to enhance the host resistance against pathogen and in mediating stress responses (Proietti et al. 2013). The interplay between SA and ABA has been identified as a crucial factor for virus-induced drought tolerance (Aguilar and Lozano-Duran 2022).

**Fig. 1:**
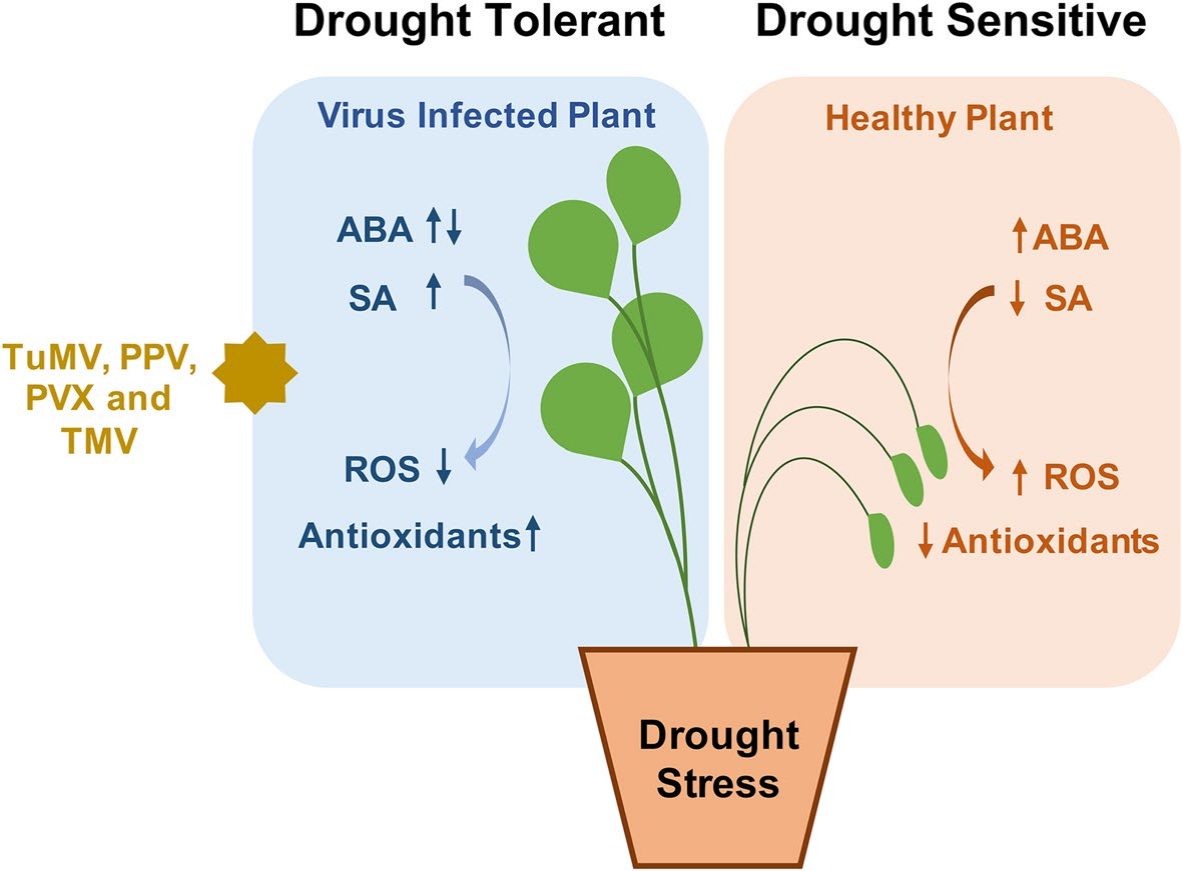
Schematic representation of virus-induced changes in plants during drought stress. Under drought, healthy plant accumulates increased abscisic acid (ABA) which leads to increased content of reactive oxygen species (ROS), leading to wilting and death of the plant (drought sensitive). However, several viruses, such as Turnip mosaic virus (TuMV), Plum pox virus (PPV), Potato virus X (PVX) and Tobacco mosaic virus (TMV) infected plants, under drought, accumulate elevated levels of salicylic acid (SA) which leads to increased content of antioxidants and reduced reactive oxygen species (ROS) content, helping plants survive under drought stress (drought tolerant)

**Fig. 2:**
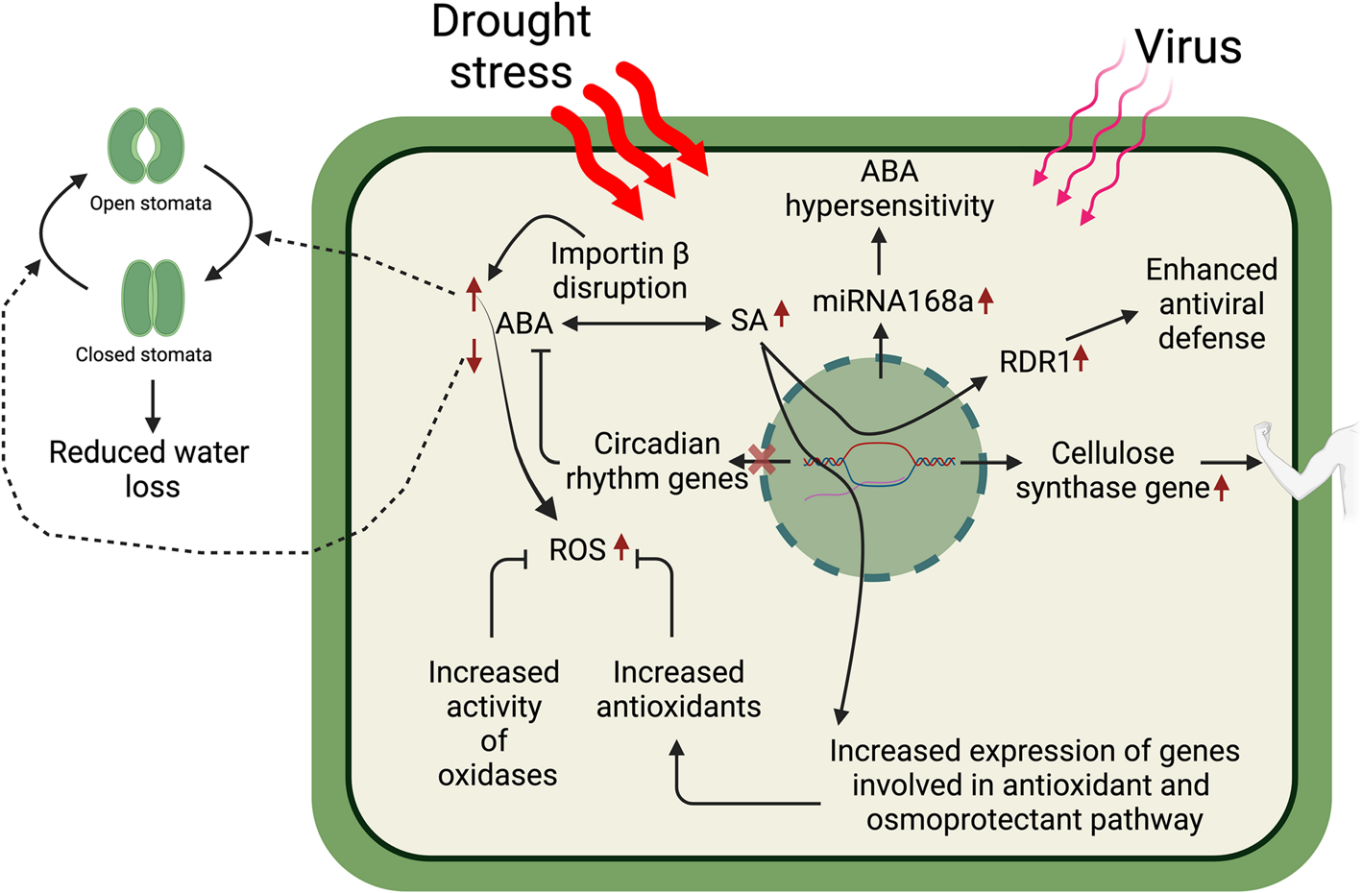
Schematic representation of various mechanisms of virus-induced drought tolerance. The level of salicylic acid (SA) is increased in virus-infected plants under drought conditions. SA content causes increased expression of genes functioning in osmoprotectant and antioxidant pathways causing high accumulation of antioxidants and osmoprotectants. Oxidases, which scavenge reactive oxygen species (ROS), also accumulate more. These compounds reduce the level of ROS. SA also induces the expression of an antiviral protein *RNA dependent RNA polymerase-1 (RDR1)* which limits the level of virus through RNA silencing. Drought and various viral suppressors of RNA silencing (VSRs) induce *miRNA168a* level which causes ABA hypersensitivity and drought tolerance. Certain viruses are reported to induce the expression of *cellulose synthase* genes leading to strengthening the cell and drought resilience. Disrupting the nucleo-cytoplasmic transport proteins, such as importin β1, leads to ABA hypersensitivity, which further direct closing of stomata and reducing water loss, thus improving survival under drought. While inhibiting certain genes of circadian rhythm also improves drought resilience by making the plant hypersusceptible to ABA. However, these effects may vary for different pathosystems

ABA functions in abiotic stress tolerance and can positively or negatively affect the plant during biotic and abiotic stresses (Denancé et al. 2013) (Fig. 1). Drought stress is known to cause increased accumulation of ABA which in turn causes closure of stomata and water loss (Lim et al. 2015). However, prolonged exposure to ABA can cause damage to the cells through increased Reactive Oxygen Species (ROS) production (Li et al. 2022). The response to abiotic stress is frequently governed by the coordinated action of interconnected signaling pathways, including both ABA-dependent and ABA-independent mechanisms. In tobacco plants, infection with TMV significantly elevates the concentration of ABA (Whenham et al. 1985). SA plays a central role in biotic stress and has been shown to be induced by various pathogens (Prakash et al. 2022; Malavika et al. 2023). SA is also induced during drought stress in many plants such as rice, wheat, and *A. thaliana* (Kang et al. 2013; Miura et al. 2013; Munsif et al. 2022). There are several reports showing that virus infected-drought tolerant plants have high levels of SA compared with non-drought stressed/healthy plants (Aguilar et al. 2017; Prakash et al. 2023; Malavika et al. 2023) (Fig. 1). Thus, hypothetically it’s possible that when the plant is suffering from both drought stress and virus infection, ABA level is reduced (to avoid the buildup of ROS) and the SA level is increased (Fig. 2). For instance, the level of plant hormones changes, and particularly, Prakash et al. showed that the level of SA is induced in the transgenic *A. thaliana* plants overexpressing *Potyvirus Turnip mosaic virus* (TuMV) 6K2 protein and in *N. benthamiana* plants infected with a viral construct overexpressing TuMV 6K2 protein under drought condition (Prakash et al. 2023). However, the level of ABA was not changed in both the cases, neither when infected with TuMV nor in TuMV-6K2 transgenic *A. thaliana*. *A. thaliana* plants co-infected with PPV and PVX showed better drought tolerance compared with the single infection and had more SA level (Aguilar et al. 2017). However, SA level is not changed in drought tolerant Arabidopsis infected with TYLCV, suggesting the involvement of other effectors in providing drought tolerance (Corrales-Gutierrez et al. 2020). Under abiotic stress, SA also has been shown to modulate plant metabolic processes, such as increased Proline metabolism (Khan et al. 2015). Proline acts as an osmoprotectant, helps in maintaining cell’s turgor pressure and deactivates ROS (Aguilar et al. 2017; Aguilar and Lozano-Duran 2022). CMV 2b protein (a suppressor of gene silencing) has been shown to induce drought tolerance in *A. thaliana* by interfering with ABA signaling via affecting host’s RNA silencing mechanism (Westwood et al. 2013; Carr 2017). In addition to providing tolerance to drought, CMV infection also improved reproductive fitness of *N. benthamiana* (Moreno et al. 2022). SA induces the expression of *RNA dependent RNA polymerase-1 (RDR1),* a host antiviral protein which functions in generating small-interfering RNA (siRNA)*,* which might help in the downregulation of the genes which otherwise are responsible for the drought susceptibility in plants (Prakash et al. 2017; Ragunathan et al. 2021). RDR1 is also known to induce the expression of many defense related genes (Prakash et al. 2020). Thus, it would be interesting to see if SA mediated induction of *RDR1* could contribute to providing drought tolerance by affecting the expression of defense related genes. Interestingly, *RDR1* promoter of various plant species possess binding sites for MYB family of transcription factors which are involved in disease resistance and abiotic stress tolerance further providing clue for the potential role of RDR1 protein in plant drought tolerance which needs to be experimentally proven (Katiyar et al. 2012; Prakash and Chakraborty 2019).

## Enhanced accumulation of Antioxidants and Osmoprotectants

Viral infections in plants can trigger a cascade of responses aimed directly or indirectly at combating the drought stress. One such response involves the activation of defense mechanisms by accumulation of antioxidants and osmoprotectants that also happen to confer drought tolerance. Osmoprotectants and antioxidants help plants fight abiotic stresses (Singh et al. 2015). Antioxidants play a crucial role in scavenging ROS generated during stress conditions such as drought and viral infections. ROS can cause oxidative damage to cellular components, leading to cell death and tissue damage. Therefore, the accumulation of antioxidants helps to mitigate this damage and maintain cellular homeostasis. SA is also known to induce the genes involved in increasing antioxidants (Dat et al. 1998; Aguilar and Lozano-Duran 2022; Yadav et al. 2024). Király et al. showed that the resistance in *N. edwardsonii* ‘Columbia’ against TMV and *Alphanecrovirus* (RNA virus) *Tobacco necrosis virus* (TNV) was also associated with enhanced level of antioxidant glutathione and glutathione-S-transferase enzymes (Király et al. 2024). Exogenous application of SA also leads to increased activity of antioxidant enzymes and reduced H_2_O_2_ (Yadav et al. 2024) (Fig. 2). It would be interesting to check the effect of drought on these plants in the presence and absence of virus infection. Xu et al. showed that BMV and CMV induced drought tolerance was accompanied by increased accumulation of anthocyanins, tocopherols and ascorbic acid which are well known antioxidants (Xu et al. 2008). BMV*,* CMV and *Tobamovirus Yellow tail flower mild mottle virus* (YTMMV) infected plants also accumulated elevated levels of sugars such as trehalose, fructose, sucrose, glucose and other osmoprotectants such as putrescine and proline (Xu et al. 2008; Dastogeer et al. 2018).

## Reducing reactive oxygen species (ROS)

Oxidative stress is a common consequence of both abiotic and biotic stress. (Hasanuzzaman et al. 2013; Hernández et al. 2016). By promoting antioxidant systems, SA helps the plant to mitigate the damage caused by ROS, which is produced under stress conditions (Figs. 1 and 2). Treating leaves with methyl viologen (paraquat) causes photobleaching, which in turn is caused by the production of superoxide ions and oxidation of molecular oxygen (Vaughn and Duke 1983). The level of ROS production is reduced in virus infection. For example, YTMMV-infected *N. benthamiana* showed reduced photobleaching compared to non-infected plants subjected to drought (Dastogeer et al. 2018). More photobleaching suggests increased ROS accumulation. Accumulation of ROS is reduced by several oxidases such as catalase, peroxidases, and polyphenol oxidases. Indeed, YTMMV infection caused a steady increase in catalase, peroxidase, and polyphenol oxidase activity under drought condition which might be the reason for the reduced photobleaching in YTMMV infected *N. benthamiana* leaf discs during drought compared to non-infected leaf discs (Dastogeer et al. 2018). PVX infection also increases tolerance to oxidative stress in *N. benthamiana* (Shabala et al. 2010, 2011a, b).

## Affecting players of RNA silencing/miRNA pathway

RNA silencing is a natural antiviral defense mechanism of plants. Viruses can be both the inducers and targets of RNA silencing machinery. Viruses have evolved specialized proteins called viral suppressors of RNA silencing (VSRs) to counter host defense machinery (Anandalakshmi et al. 1998). These VSRs block host RNA silencing machinery through interaction with RNA silencing machinery components (Basu et al. 2014). VSRs have acquired other functions also, such as, CMV 2b protein interfere with ABA pathway and provide drought tolerance in *A. thaliana* (Westwood et al. 2013). Another mechanism through which viruses influence drought tolerance is by altering the host's miRNA profile. For example, Foveavirus (RNA virus) *Grapevine rupestris stem pitting associated virus *(GRSPaV) infected grapevine had altered profile of miRNA involved in water stress compared to virus free plants (Pantaleo et al. 2016). *miRNA168* is a stress inducible miRNA and the expression of this miRNA is altered in drought stress (Zhou et al. 2010). *miRNA 168a* targets *Argonaute-1 (AGO1)* and plants expressing *miRNA168a* are tolerant to drought and hypersensitive to ABA, similar to *ago1* mutant *A. thaliana* (Li et al. 2012) (Fig. 2). Various VSRs cause enhanced accumulation of *miRNA168a,* thus affecting AGO1 function and drought (Várallyay and Havelda 2013). Begomovirus *South African cassava mosaic virus* (SACMV) infection in cassava landraces show differential expression of *miRNA168a* affecting the susceptibility and tolerance of the host (Bizabani et al. 2021).

## Altering expression of genes involved in circadian rhythm

Circadian rhythms in plants govern various physiological and developmental processes, including leaf movement, stomatal conductance, and flowering time, helping them adapt to environmental changes over a 24-h cycle (McClung 2006). The role of circadian rhythm has been attributed to provide tolerance to abiotic stresses including drought (Grundy et al. 2015). During infection, circadian rhythm also affects the host traits which increases fitness of host and parasite (Westwood et al. 2019). Virus infected plants under drought stress show altered transcription of several circadian rhythm related genes. For example, Gonzalez et al. showed that TuMV infected *A. thaliana* under drought stress shows reduced expression of genes related to circadian rhythm such as *PSEUDO-RESPONSE REGULATOR 5 (PRR5)* and *FLAVIN-BINDING KELCH REPEAT FBOX 1 (KFK1)* (González et al. 2021). PRR5 acts as a transcriptional repressor of MYB transcription factors involved in circadian rhythm, *LATE ELONGATED HYPOCOTYL 1(LHY1)* and *CIRCADIAN CLOCK ASSOCIATED 1 (CCA1)* (González et al. 2021). Reduced expression of *PRR5* leads to increased expression of *LHY1* which induces the accumulation of ABA responsive genes, providing drought tolerance (Westwood et al. 2013; Adams et al. 2018; González et al. 2021). Another report showed that *prr5,7,9* triple mutant shows enhanced drought tolerance (Nakamichi et al. 2009). KFK1 positively regulates the expression of *CONSTANS (CO)* thus reduced expression of *KFK1* leads to reduced *CO* expression*.* Reduced expression of a *CO*-like gene has been linked with enhanced drought tolerance in rice (Liu et al. 2016). Taken together, these observations suggest that during drought conditions, virus infection modulates the expression of genes involved in circadian rhythm to provide drought tolerance (Fig. 2).

## Affecting expression of genes involved in nucleocytoplasmic trafficking

The interplay between plant viruses, nucleocytoplasmic trafficking, and drought stress has been a subject of recent research. Krichevsky et al. (2006) highlighted the significance of plant viruses manipulating cellular machinery through interactions with host factors involved in nucleocytoplasmic transport to establish infection and spread within the plant host (Krichevsky et al. 2006). This observation is later supported by Gonzalez et al. (2021), who demonstrated that TuMV-infected *A. thaliana* plants subjected to drought stress had reduced expression of genes involved in nucleocytoplasmic trafficking (González et al. 2021). In addition, Yang et al. (2017) showed enhanced drought tolerance after disrupting the genes functioning in nucleocytoplasmic transport (Yang et al. 2017). Consistent with that result Luo et al. (2013) demonstrated that impairing *A. thaliana* importin β1 increased drought tolerance by promoting stomata closure and reducing water loss (Luo et al. 2013). The importance of nucleocytoplasmic transport in virus-infected plants subjected to drought stress is also emphasized by another study which suggested that nucleocytoplasmic trafficking plays a key role in plant disease resistance, hormone signaling, and development (Dong et al. 2006). In conclusion, the manipulation of nucleocytoplasmic trafficking by plant viruses and its impact on drought stress response in infected plants is a complex and critical area of research and more research is still needed to understand the intricate mechanisms underlying the role of nucleocytoplasmic trafficking in virus-infected plants under drought stress conditions.

## Induced expression of genes for cellulose biosynthesis

Cellulose is a crucial part of the plant cell wall. Cellulose provides an important role in strengthening the plant cells under extreme abiotic conditions such as drought and osmotic stresses (Chen et al. 2005). Decreased content of cellulose has been reported during drought stress indicating the involvement of cellulose in the plant's response to water deficit conditions (van der Weijde et al. 2017; Teixeira et al. 2020). Moreover, increasing cellulose content has been associated with tolerance to osmotic and drought stress in plants (Li et al. 2019). Virus infection affects the cellulose biosynthesis, which is shown by several reports. For instance, Seo et al. (2018) reported that TYLCV infection affects cellulose and hemicellulose biosynthesis (Seo et al. 2018). While Mirzayeva et al. (2023) showed that in tomato plants, tolerance to drought was achieved by TYLCV and Crinivirus (RNA virus) *Tomato chlorosis virus* (ToCV) mediated induced expression of cellulose synthase genes *Ces-A2, Csl-D3,2,* and *Csl-D3,1* (Mirzayeva et al. 2023). Altogether, these reports suggest the importance of cellulose in providing tolerance to drought during virus infection and understanding the relationship between virus infection and cellulose biosynthesis might have potential to engineer virus-resistant and drought-tolerant plants (Fig. 2).

## Affecting the expression of heat shock proteins (HSPs)

Plant HSPs play a vital role under both biotic and abiotic stresses (Moshe et al. 2016; Kumar et al. 2023b, a). Several HSPs are induced during the heat stress in plants (Kumar et al. 2015, 2016, 2020). The role of plant HSPs has been implicated in providing tolerance to biotic stresses, including responses to viral infections (ul Haq et al. 2019). Expression of HSPs is induced in heat stress (Usman et al. 2017). Research highlighted the involvement of HSPs in the establishment of virus infections, such as TYLCV and PVY (Gorovits and Czosnek 2017; Makarova et al. 2018). PVY infected potato subjected to heat stress showed altered expression of *HSP70* and *HSP90* genes (Makarova et al. 2018). Moreover, researchers observed ambiguous role of HSP70 during virus infection, resulting in both positive and negative effects in host-virus interaction, suggesting a complex relationship between HSP70 and virus infection (Hyskova et al. 2021). Reports suggest that HSP70 interacts with TuMV RNA dependent RNA polymerase (NIb) during virus replication (Dufresne et al. 2008). In addition, it has been demonstrated that PVY can induce the expression of *HSP70* during the development of infection, further emphasizing the link between virus infection and *HSP* induction in plants (Kozieł et al. 2021). Moreover, tomato plants show high mortality and severe growth retardation when HSP70 was silenced, suggesting positive role of HSP70 under drought stress (Aghaie and Tafreshi 2020).

Reduced expression of *HSP90*, *HSP70*, and three *heat stress transcription factors (HSFs)* were reported during TYLCV infection in drought stressed plants (Mishra et al. 2022). Similarly, Shteinberg et al. (2021) found that the presence of TYLCV in plants caused a down-regulation of stress response proteins, including *HSP90* and *HSP70*, in response to drought (Shteinberg et al. 2021). However, the research by Gorovits & Czosnek (2017) revealed that TYLCV does not induce the expression of *HSP70* in plants (Gorovits and Czosnek 2017). Overall, the evidence from these studies suggests that under drought conditions, TYLCV infected plants exhibit either reduced or unaltered expression of *HSP90*, *HSP70*, and *HSFs*, indicating a potential modulation of HSPs and HSFs in response to viral infection in certain plants-virus interactions and drought stress in plants. The direct link between HSPs and virus-induced drought tolerance is lacking, and further research is needed to understand the role of HSPs in positively or negatively regulating the virus-induced drought tolerance.

## Conclusion and future directions

Viruses are obligate parasites with a small genome size and limited protein coding capacity. Upon infection under normal conditions, viruses hijack and manipulate host cellular machinery for successful infection and propagation in the host plants. Under drought conditions, the virus infected host plant is further challenged to cope and survive dual stress conditions. Under dual stress conditions, the host machinery redirects its cellular resources to combat drought stress. This rerouting of resources may ultimately put pressure on virus fitness in the host. This resource shortage may function as a feedback loop and ultimately lead the viral pathogen to shift its nature from parasitism to mutualism and activate host processes responsible for drought tolerance. It is possible that when host plant is under drought stress, virus infection induces, directly or indirectly, yet unknown plant effectors which ultimately are responsible for the drought tolerance phenotype, however these effectors are yet to be discovered. In our present understanding, there are various ways through which viruses provide drought tolerance, such as by affecting transcriptome and metabolites, increasing the content of osmoprotectants and antioxidants etc. Further exploration of these mechanisms could be an excellent, sustainable and ecofriendly way to generate drought tolerant crops in future which needs more interdisciplinary collaboration and innovative research approach. Scientists would also need to ensure safe and sustainable integration of any outcome into agricultural practices. There is enormous potential for establishing resilient and sustainable agriculture system by deeply understanding the mechanisms of plant-virus interactions.

## Data Availability

Not applicable.

## Abbreviations

BMV: Brome mosaic virus
CMV: Cucumber mosaic virus
TMV: Tobacco mosaic virus
TRV: Tobacco rattle virus
PVX: Potato virus X
PPV: Plum pox virus
TYLCSV: Tomato yellow leaf curl Sardinia virus
TYLCV: Tomato yellow leaf curl virus
ABA: Abscisic acid
SA: Salicylic acid
TNV: Tobacco necrosis virus
RDR1: RNA dependent RNA polymerase-1
YTMMV: Yellow tail flower mild mottle virus
ROS: Reactive oxygen species
VSRs: Viral suppressors of RNA silencing
GRSPaV: Grapevine rupestris stem pitting associated virus
AGO1: Argonaute-1
SACMV: South African cassava mosaic virus
PRR5: PSEUDO-RESPONSE REGULATOR 5
KFK1: FLAVIN-BINDING KELCH REPEAT FBOX 1
LHY1: LATE ELONGATED HYPOCOTYL 1
CCA1: CIRCADIAN CLOCK ASSOCIATED 1
CO: CONSTANS
ToCV: Tomato chlorosis virus
HSPs: Heat shock proteins
HSFs: Heat stress transcription factors
